# Predictors of advanced chronic kidney disease in infancy after definitive vesicoamniotic shunting for congenital lower urinary tract obstruction

**DOI:** 10.3389/fped.2022.977717

**Published:** 2022-10-14

**Authors:** Chryso Pefkaros Katsoufis, Marissa DeFreitas, Jessica Leuchter, Wacharee Seeherunvong, Jayanthi Chandar, Carolyn Abitbol

**Affiliations:** Department of Pediatrics, Division of Pediatric Nephrology, University of Miami Miller School of Medicine/Holtz Children's Hospital, Miami, FL, United States

**Keywords:** congenital lower urinary tract obstruction, bladder outlet obstruction, vesicoamniotic shunt, nadir serum creatinine, chronic kidney disease, proteinuria

## Abstract

**Background:**

Severe congenital lower urinary tract obstruction (cLUTO) is associated with poor postnatal outcomes, including chronic and end stage kidney disease, and high mortality. Studies of the impact of fetal intervention through vesicoamniotic shunting are marred by a device malfunction rate of up to 60%. In this study, we delineate the postnatal course and infant kidney function following definitive urinary diversion *in utero*.

**Materials and Methods:**

This is a retrospective, single-center cohort study of 16 male infants who survived the fetal intervention to birth, from 2010 to 2014 at a single center. All had patent shunts in place at birth. Perinatal and biochemical characteristics were collected with patients followed for one year, or until demise, with serial measures of serum creatinine (SCr) and serum cystatin C (CysC).

**Results:**

Of the 16 males, 81% were non-white (38% black, 43% Hispanic). Shunts were placed at a median of 20 weeks (IQR 19,23) gestation, with median fetal bladder volume of 39 cm^3^ (IQR 9.9,65). All neonates were born preterm [median 34 weeks (IQR 31,35)] and the majority with low birth weight [median 2340 grams (1,895, 2,600)]. 63% required positive pressure ventilation. Advanced chronic kidney disease stage 4–5 at 1 year of age was predicted by neonatal characteristics: peak SCr ≥2 mg/dl, time to peak SCr > 6 days, discharge SCr ≥1.0 mg/dl, CysC ≥2.5 mg/l, urine protein:creatinine ≥4.8 mg/mg, urine microalbumin:creatinine ≥2.2 mg/mg. In infancy, a nadir SCr ≥0.5 mg/dl occurring before 160 days (5.3 months) of age was also predictive of advanced chronic kidney disease stage 4–5 at 1 year. Three patients died in the neonatal period, with 1 receiving kidney replacement therapy (KRT). Three additional patients required KRT before 12 months of age.

**Conclusions:**

Even with definitive vesicoamniotic shunting for cLUTO, postnatal morbidity and mortality remain high, emphasizing the role of renal dysplasia, in spite of urinary diversion, in postnatal kidney dysfunction. Neonatal and infant biochemical parameters exhibit distinct trends that offer families and physicians a better understanding of the prognosis of childhood kidney function.

## Introduction

The spectrum of congenital lower urinary tract obstruction (cLUTO) includes the three most common diagnoses of posterior urethral valves (PUV), urethral atresia and Eagle-Barrett syndrome, with respective prevalence rates of 64%, 32% and 4% (1). The incidence of cLUTO is estimated at 2–3 per 10,000 births, with a significantly higher prevalence in black and minority ethnic groups approximating 6–7 per 10,000 births (1). With a perinatal mortality rate as high as 120 per 1,000 isolated cases, and nearly four times that in complex cases with extrarenal anomalies (1), contemporary therapeutic interventions aim to relieve the anatomic obstruction early *in utero*. The antenatal diagnosis of fetal cLUTO is based on the identification of megacystis, with a fetal bladder diameter greater than 7 mm by screening anatomical ultrasound (2). However, megacystis up to 15 mm in the first trimester is also known to regress spontaneously in up to 43% of cases (3, 4). Therefore, the decision to proceed with fetal intervention becomes predicated on the presence of oligohydramnios, which is historically viewed as a surrogate of fetal kidney dysfunction in the third trimester (5). Even though multicenter investigation revealed a requirement of kidney replacement therapy (KRT) in a third of cLUTO patients with normal amniotic fluid volume in the second trimester (6), antenatal staging algorithms recommend deferring intervention in fetuses without oligohydramnios (7, 8). This deferral is supported by the attribution of perinatal mortality to severe oligohydramnios and resultant pulmonary hypoplasia with respiratory failure (9). Furthermore, metanalyses with systematic review inclusive of the Percutaneous shunting in Lower Urinary Tract Obstruction (PLUTO) study have yielded a survival benefit of vesicoamniotic shunt (VAS) placement only to those fetuses with the poorest prognosis based on amniotic fluid volume and urine biochemistry (10). However, these analyses are confounded by a mechanical complication rate of 20%–60% (4). As a result, the discussion remains open as to whether VAS may provide any benefit to kidney function.

In spite of advancements in prenatal diagnosis and novel fetal interventions such as VAS and cystoscopy, kidney functional outcomes have not shown to be significantly impacted in cLUTO (11). This supports the concept that the primary burden of cLUTO-related chronic kidney disease (CKD) lies is the dysplastic kidney parenchyma. With some authors inferring that fetal intervention may be facilitating the survival of the most severe cases while increasing the prevalence of overall childhood CKD (2), it remains of great importance to understand the clinical biomarkers that facilitate the care of these infants and the counseling of their families. With all of its known shortcomings, serum creatinine (SCr) remains the most widely used surrogate of current kidney function and predictor of future kidney function (12). Infant trends in SCr have been characterized in males with posterior urethral valves with prognostic value in predicting future kidney dysfunction in childhood (13). However, the significance of neonatal biochemistry on these trends is not as well studied, nor is the impact of VAS. Limited data is published regarding the role of proteinuria and/or albuminuria as predictive biomarkers in cLUTO, with even less understood regarding serum Cystatin C (CysC), though each of these biomarkers has been used in varying degrees to predict CKD progression in children in general (14, 15).

This study aims to delineate the impact of VAS on a cohort of infants with severe cLUTO, who were all born with mechanically functional shunts. The trends in biochemical characteristics are compared between those children with CKD stage 1–3 vs. 4–5 at one year of life, to guide future medical practice and the approach to KRT in this vulnerable population.

## Materials and methods

With institutional review board approval, a retrospective cohort analysis of all infants born live following fetal vesicoamniotic shunting from January 2010 through December 2014 was performed at a single center. All infants were diagnosed with cLUTO antenatally, based on ultrasonographic evidence of megacystis with or without bilateral hydronephrosis. The “keyhole” sign of the dilated proximal urethra was visualized in 12 of 16 fetuses. Fifteen of 16 mothers consented to vesicocentesis for fetal urine electrolyte analysis prior to VAS placement. All VAS were placed percutaneously by ultrasound-guidance, by a single operator with expertise in maternal-fetal medicine. Consent for fetal VAS placement was obtained independently by the perinatologist, following a shared-decision making approach with the mother. The VAS used in all subjects was the combination of a standard double pig-tail catheter and an atrial septal occluder/double-disk device (16). All infants included in the study were born live with a functional VAS in place, draining urine. Because the intent was to investigate the immediate postnatal characteristics, no infants were excluded for subsequent development of sepsis or systemic disease. In addition, because extra-renal anomalies are not uncommon in the spectrum of cLUTO, no infants were excluded for these findings.

Perinatal characteristics were recorded, including: gestational age at cLUTO diagnosis, VAS placement and birth, bladder volume and presence of oligohydramnios at diagnosis, neonatal weight and length, need for positive pressure ventilation and intensive care length of stay. Fetal urine electrolytes were analyzed from the initial vesicocentesis, consistent with the center protocol at that time, with the following considered to be unfavorable: sodium >100 mmol/L, calcium >8 mg/L, chloride >90 mmol/L, osmolarity >200 mOsm/L, β-2 microglobulin >6 mg/L, protein >20 mg/dL (17). The biochemical markers collected in the study period included SCr at its neonatal peak or maximum rise after birth, at discharge from the intensive care unit at a time deemed appropriate by the medical care team, and at 1 to 3-month intervals throughout the first year of life to determine the nadir SCr or lowest recorded level. SCr was measured by the enzymatic method. CysC was assayed during the neonatal hospitalization within 3 weeks of birth, and at 3-month intervals throughout the first year of life. CysC levels were measured by the particle-enhanced immunonephelometric immunoassay (Dade-Behring, Deerfield, Illinois). Random urine samples were profiled for proteinuria and albuminuria during the neonatal intensive care hospitalization, prior to discharge.

Patients were categorized into two subgroups according to their stage of CKD at 1 year of life. The first group, CKD 1–3, had an estimated glomerular filtration rate (eGFR) ≥30 ml/min/1.73 m^2^ by 12 months of age. The second group with advanced CKD, CKD 4–5, had an eGFR <30 ml/min/1.73 m^2^ by 12 months of age. eGFR was calculated from SCr using the revised Schwartz equation from the CKiD report (18): eGFR_cr_ = 41.3 [height (meters)]/SCr (mg/dl). It was also calculated from CysC as recommended in infants and subjects with low muscle mass (19): eGFR_cys_ = 70.69(CysC)^−0.931^. While absolute values of eGFR may have differed, the CKD stage was consistent between the two measurement techniques in all patients.

All postnatal biochemical parameters were collected based on provider recommendation, in accordance with center practice pattern. This includes the use of Cystatin C routinely, given the authors' previously published study of this biomarker in the neonatal population (19).

Data sets were tested for normality with the D'Agostino and Pearson omnibus normality test. Due to the small cohort size, continuous variable data are expressed as the median with interquartile range (IQR) (25th,75th percentile) as appropriate. Intergroup comparisons were tested with Mann-Whitney U for nonparametric data. Proportional differences between the 2 groups were tested with the Fisher exact test with odds ratios and 95% confidence intervals. The odds ratios were then graphed in a Forest plot. Receiver operating characteristic (ROC) and area under the curve (AUC) analyses were used to assess the sensitivity and specificity of perinatal and biochemical markers to predict the early progression to advanced CKD by 1 year of age. The threshold was taken as the value of the marker with the highest sensitivity at a specificity ≥80% with a significant likelihood ratio. Simple linear regression was used to determine the correlation between SCr or CysC over time, in each subgroup. Statistical analyses were performed using GraphPad Prism (GraphPad Software, Inc, La Jolla, California). A *p* value < 0.05 was considered significant, and all analyses were two-tailed.

The university-based institutional review board waved consent for this retrospective analysis of infants born live at our center.

## Results

Of the 16 infants born live during the study period, all had a functioning VAS *in situ* at birth. Table 1 provides the demographic characteristics, including race/ethnicity, primary diagnosis/phenotype and CKD stage at 1 year of life. The mothers were predominantly non-white (81%) with 6 (38%) identifying as black, African American or Afro-Caribbean, and 7 (43%) identifying as Hispanic. The primary diagnosis was posterior urethral valves in the majority, as expected. Six of the infants had more significant urethral stenosis or atresia, while 1 infant with severe megacystis but a normal urethral caliber was presumed to have megacystis microcolon intestinal hypoperistalsis syndrome (MMIHS). Of the 16 with various primary diagnoses, 6 (38%) had a lax abdomen phenotype. Four of the 6 had the constellation of findings consistent with Eagle-Barrett syndrome, while an additional two were variants without the full triad that includes undescended testicles. Ultimately, the majority of infants (*N* = 10, 62%) developed advanced CKD stage 4–5 in the first year of life. Three infants died during the neonatal period, either within the first month of life or during the neonatal intensive care hospitalization. Two had comorbid, extrarenal anomalies and succumbed to complications of sepsis: one at 3 days of age and the other at 54 days of age on KRT. The third infant died of urosepsis, following discharge from the neonatal hospitalization with optimistic renal function (discharge SCr 0.28 mg/dl, CysC 1.43 mg/L).

**Table 1:** Patient demographics, *N* = 16.


Race, *N* (%)
Caucasian	3 (19%)
African American	3 (19%)
Afro-Caribbean	3 (19%)
Hispanic	7 (43%)
Diagnosis, *N* (%)	
Posterior Urethral Valves	9 (56%)
Urethral Stenosis/Atresia	6 (38%)
Megacystis	1 (6%)
Lax Abdomen Phenotype, *N* (%)	*6* (*38%)*
Eagle-Barrett Syndrome	4 (25%)
Variant	2 (13%)
CKD Stage at 1 Year, *N* (%)	
Stage 1–3	6 (38%)
Stage 4–5	10 (62%)

Perinatal characteristics are summarized in Table 2. In spite of the difference in kidney function outcomes at 1 year of life, there is little distinction between the two groups at the time of diagnosis. All patients were diagnosed in the second trimester at a median of 18 weeks of gestation. Oligohydramnios was common with bladder volume estimated at a median of 39 cm^3^ (range 3.6 to 90.7 cm^3^). Based on the cLUTO staging system proposed by Fontanella et al (20)., all of the fetuses would be classified as “Severe” by bladder volume ≥5.4 cm^3^ or oligohydramnios prior to 20 weeks. VAS was inserted at a median gestational age of 20 weeks. Preterm birth before 37 weeks gestation was universal at a median of 34 weeks. The CKD 4–5 group trended towards more significant prematurity with a median gestational age of 34 weeks compared to 36 weeks in the CKD 1–3 group, with a *p* value equal to 0.05. In spite of an increase in amniotic fluid volume following VAS placement, the need for positive pressure ventilation remained common for 63% of the cohort, during the neonatal intensive care stay. However, of the 14 infants who survived to hospital discharge, none were dependent on supplemental oxygen by that time. Neonatal intensive care unit length of stay was statistically similar between the two groups but trended towards longer in the CKD 4–5 group.

**Table 2:** Perinatal characteristics.

	ALL *(16)*	CKD 1-3 *(6)*	CKD 4-5 *(10)*
GA, *weeks* – Diagnosis	18 (16,22)	18 (16,22)	18 (16,21)
Bladder Volume, *cm^3^* – Diagnosis	39 (9.9,65)	39 (16,58)	37 (6.7,75)
Oligohydramnios, *N* (%) – Diagnosis	13 (81%)	4 (67%)	9 (90%)
GA, *weeks* – Shunt Placement	20 (19,23)	21 (19,27)	20 (18,22)
GA, *weeks* – Birth	34 (31,35)	36 (33,36)*	34 (31,34)*
Birth Weight, *grams*	2,340 (1895,2600)	2,438 (2125,2888)	2,275 (1760,2480)
Birth Weight, *Z-score*	0.26 (−0.32,1.2)	0.26 (−1,0.78)	0.26 (−0.31,1.3)
Birth Length, *cm*	42 (36,44)	44 (42,49)	44 (42,46)
Birth Length, *Z-score*	0.2 (−0.65,0.59)	0.14 (−2.2,0.89)	0.32 (−0.35,0.63)
Positive Pressure Ventilation, *N* (%)	10 (63%)	2 (33%)	8 (80%)
Neonatal Length of Stay, *days*	46 (27,64)	32 (25,60)	54 (43,72)

GA, Gestational age; Data presented as median (1st Quartile, 3rd Quartile) unless otherwise specified *p = 0.05.

**Figure 1:**
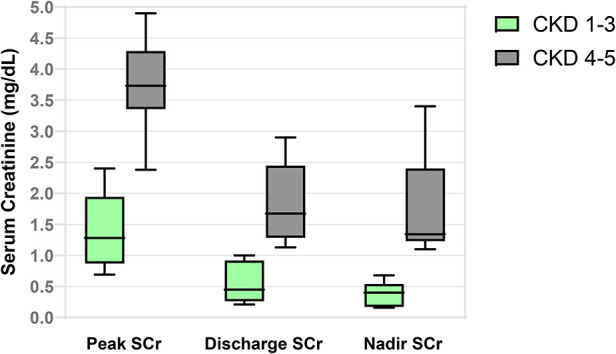
Early milestones in serum creatinine based on CKD stage at 1 year.

Biochemical characteristics were measured throughout the neonatal course. SCr was measured nearly daily in the first 2 weeks of life and then at least every 3 days until neonatal discharge. Peak SCr was significantly higher in the CKD 4–5 group at a median value of 3.7 mg/dl compared to 1.3 mg/dl in the CKD 1–3 group. Furthermore, this higher peak was reached later in time at a median of 8 days of life. ROC-AUC analyses revealed a likelihood ratio (LR) of 5.3 for CKD 4–5 in the first year of life when neonatal SCr continued to rise for more than 6 days after birth. In addition, when the neonatal SCr rose above 2.0 mg/dl, there was an LR of 6 for CKD 4–5. In concordance with the natural history of a downturn in SCr as peripheral vascular resistance decreases and systemic blood pressure increases postnatally (21), the SCr at discharge from the intensive care unit was lower than the peak in both groups but remained significantly higher in the CKD 4–5 group compared to the CKD 1–3 group. Accordingly, a discharge SCr ≥1 mg/dl carried a LR of 6 for CKD 4–5 in the first year. Neonatal CysC was collected at a median of 2 days of life (IQR 2,4). Neonatal CysC was significantly higher in the CKD 4–5 group, with an LR of 5 when measured at 2.5 mg/L or more. Neonatal proteinuria was significantly elevated throughout this cLUTO cohort. The random urine total protein:creatinine ratio was significantly higher in the patients who would go on to have CKD 4–5 at 1 year of life, with a median of 7.9 mg/mg compared to 2.4 mg/mg in the CKD 1–3 group. Interestingly, the proportion of albuminuria was consistently and similarly approximated at 40%–45% of the total proteinuria across the groups. These neonatal biochemical characteristics, along with their predictive power to associate with CKD 4–5 in the first year of life, are summarized in Table 3, Figure 1 and Table 4.

**Table 3:** Biochemical characteristics.

	Peak SCr***	Time to Peak SCr***	Discharge SCr***	Neonatal CysC*	Neonatal U *p*/c*	Neonatal U m/c*	Nadir SCr**	Time to Nadir SCr**
ALL	3.4 (1.6,4)	7 (2,8)	1.2 (0.48,1.9)	2.5 (1.9,3.1)	5.1 (3,12)	2.2 (0.7,4.5)	1.2 (0.4,2)	84 (45,190)
CKD 1-3	1.3 (0.87,1.9)	1.5 (1,5.3)	0.45 (0.26,0.92)	1.9 (1.4,2.6)	2.4 (1.5,4.8)	1.1 (0.5,2.1)	0.4 (0.17,0.54)	257 (160,313)
CKD 4-5	3.7 (3.4,4.3)	8 (7,9)	1.7 (1.3,2.4)	3 (2.5,3.7)	7.9 (4.8,16)	3.2 (2,7.4)	1.3 (1.3,2.4)	50 (37,84)

Data presented as median (1st quartile, 3rd quartile); Time presented in days; CKD, Chronic kidney disease; CysC, Serum Cystatin C mg/L; SCr, Serum creatinine mg/dl; U *p*/c, Random urine protein:creatinine; U m/c, Random urine microalbumin mg/mg creatinine.

Mann Whitney comparisons of CKD 1-3 / CKD 4-5: *p < 0.05, **p < 0.01, ***p < 0.001.

**Table 4:** Receiver operating curve (ROC) / area under the curve (AUC) analyses for biochemical predictors of CKD 4-5 in the first year of life.

	AUC	Threshold	Sensitivity (%)	Specificity (%)	Likelihood Ratio	*p* Value
Peak SCr	0.98	≥2.0 mg/dl	100	83	6.0	<0.005
Time to Peak SCr	0.97	>6 days	89	83	5.3	<0.005
Discharge SCr	1.00	≥1.0 mg/dl	100	83	6.0	<0.005
Neonatal CysC	0.89	≥2.5 mg/l	83	83	5.0	<0.05
Neonatal U *p*/c	0.91	≥4.8 mg/mg	78	80	3.9	<0.05
Neonatal U m/c	0.83	≥2.2 mg/mg	75	83	4.5	<0.05
Nadir SCr	1.00	≥0.5 mg/dl	100	80	5.0	<0.005
Time to Nadir SCr	1.00	<160 days	100	80	5.0	<0.005

CKD, Chronic kidney disease; CysC, Serum Cystatin C mg/l; SCr, Serum creatinine mg/dl; U *p*/c, Random urine protein:creatinine; U m/c, Random urine microalbumin mg/mg creatinine.

The serial measurement of SCr along the course of infancy identified significant differences in the nadir SCr between the two groups. In the CKD 4–5 group, the nadir SCr was significantly higher at 1.3 mg/dl, compared to 0.4 mg/dl in the CKD 1–3 group. Furthermore, it was achieved much sooner in the CKD 4–5 group at a median of 50 days of life (IQR 37,84). Alternatively explained, patients with better long-term kidney function at 12 months of age exhibited a greater decline in SCr over a longer period of time, specifically over a median of 257 days or more than 8 months. ROC-AUC analyses reveal a LR of 5 for developing CKD 4–5 at 1 year when nadir SCr is ≥0.5 mg/dl at <160 days (5.3 months) of life. These data are summarized in Table 3, Figure 1 and Table 4, as well.

**Figure 2:**
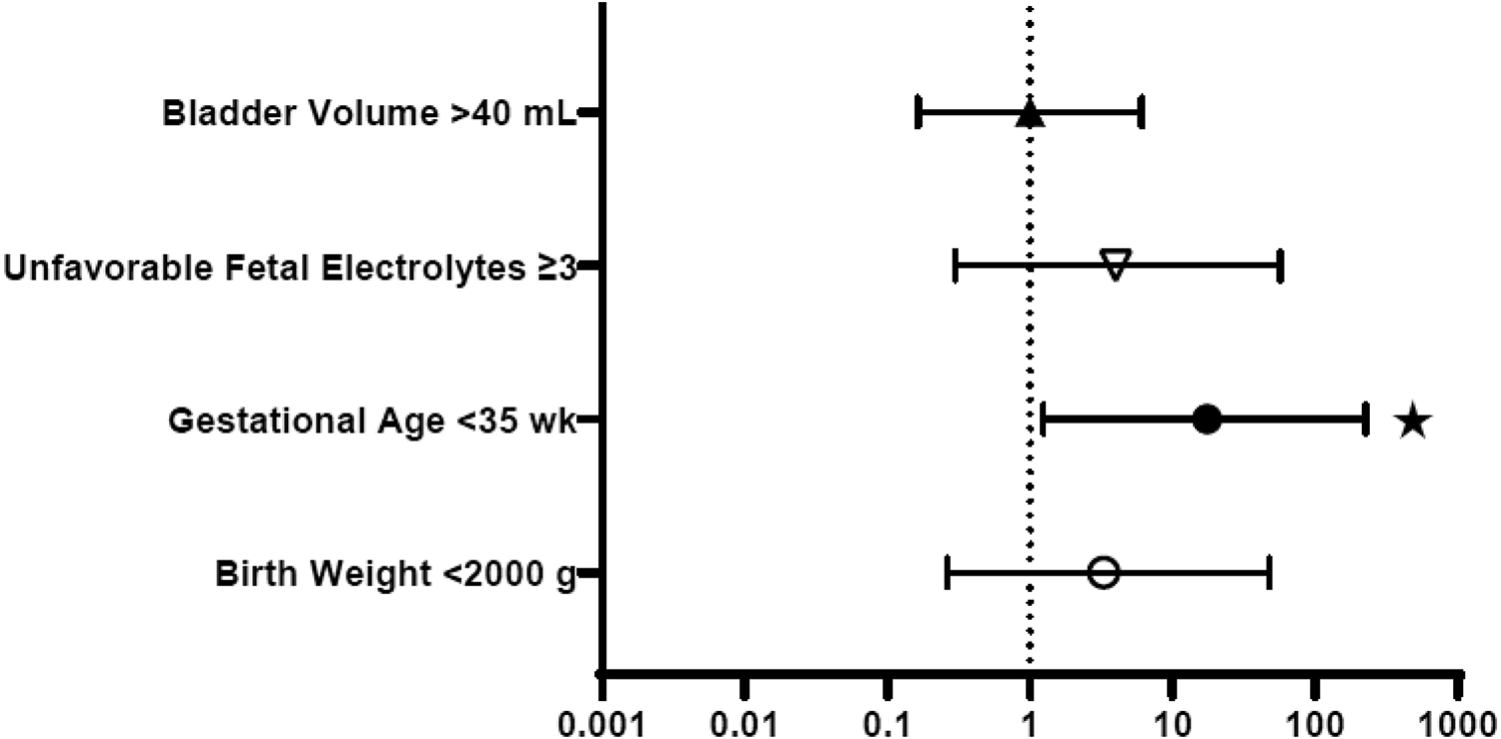
Perinatal characteristics: odds ratios for prediction of CKD stage 4–5 in the first year of life. *p < 0.05.

**Figure 3:**
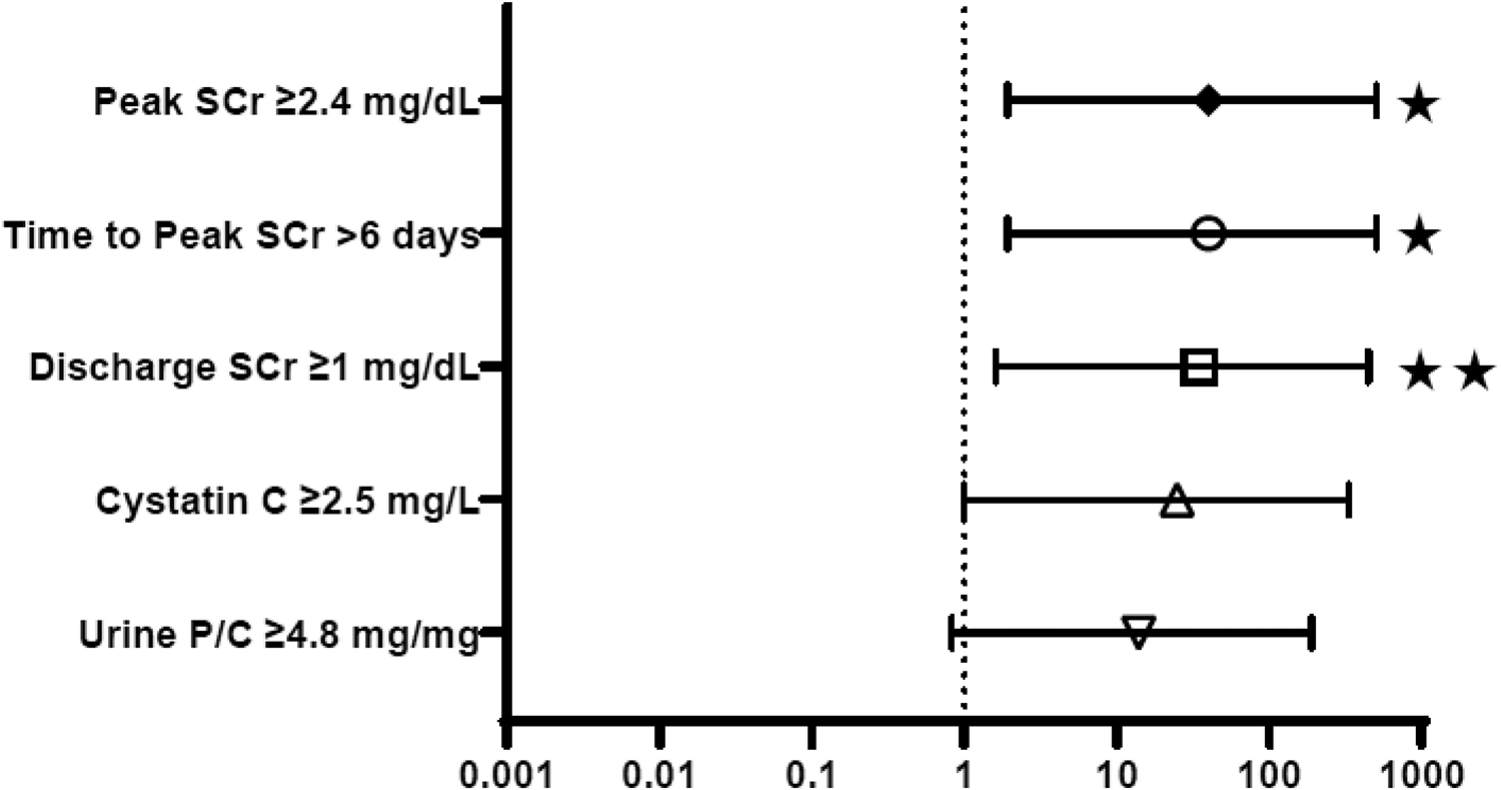
Neonatal biochemistry: odds ratios for prediction of CKD stage 4–5 in the first year of life. SCr, Serum creatinine; U *P*/C, Random urine protein:creatinine. *p < 0.05, **p < 0.005.

Figure 2 provides the odds ratios (OR) for perinatal characteristics impacting the development of CKD stage 4–5 by 12 months of age, including: bladder volume, unfavorable fetal electrolytes, gestational age and birth weight. When studied as a dichotomous variable, 3 or more unfavorable markers and initial bladder volume > 40 ml were used as the thresholds for comparison, as suggested by the scoring system for determining candidacy for fetal intervention by Nassr et al (17). As shown, only gestational age less than 35 weeks was a significant predictor of advanced CKD, with an OR of 17.5. Bladder volume >40 ml, 3 or more unfavorable fetal electrolytes and birth weight less than 2,000 grams were not. Figure 3 similarly depicts the OR for neonatal biochemical markers predicting CKD stage 4–5 at 1 year. In this model, a peak SCr of ≥2.4 mg/dl (OR 40) occurring at more than 6 days of life (OR 40), with a subsequent hospital discharge SCr of ≥1 mg/dl (OR 35) were predictive of advanced CKD 4–5 at 12 months of age. CysC and neonatal proteinuria were not significantly different between the two groups in this analysis.

**Figure 4:**
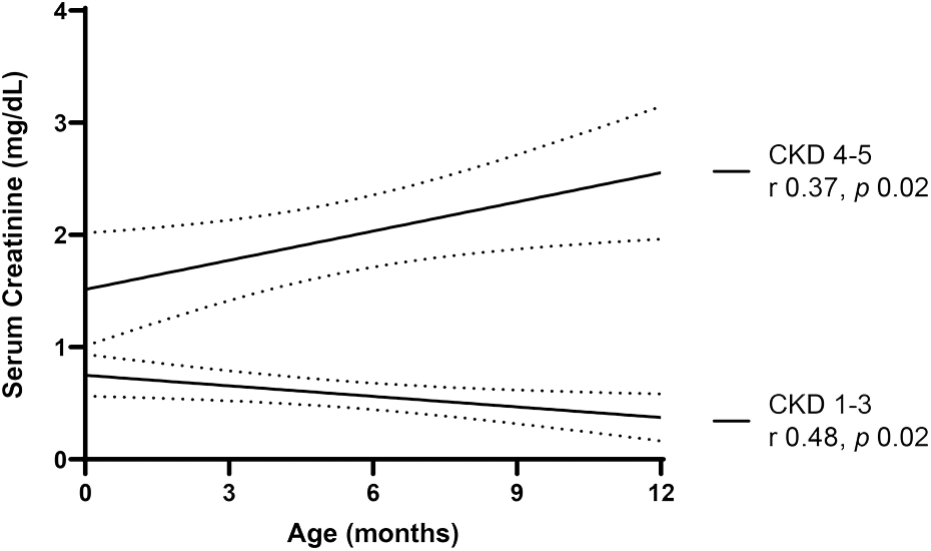
Trend in serum creatinine over the first year of life.

**Figure 5:**
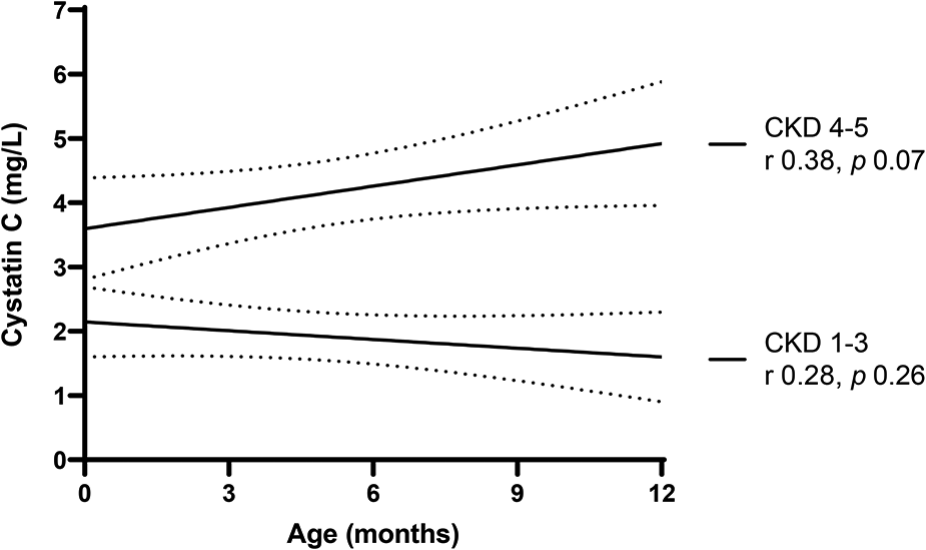
Trend in serum cystatin C over the first year of life.

Figures 4, 5 provide graphical representations of the trends in SCr and CysC over time, in each subgroup, with respective *p* values and r correlation coefficients.

Throughout the first year, patients in both groups required multiple re-hospitalizations, a median of 4 (IQR 3,5). In the CKD 4–5 group, KRT was initiated in 3 of those 10 patients, or 19% of the entire cLUTO cohort. 2 were started on peritoneal dialysis, and 1 was started on continuous kidney replacement therapy (CKRT) prior to demise during the neonatal intensive care stay. Neither of the 2 patients requiring maintenance peritoneal dialysis were initiated in the neonatal period. Lastly, of the 14 patients who survived to neonatal hospital discharge, all were able to void spontaneously without need for urethral/bladder catheterization or vesicostomy, at that time.

## Discussion

This single center cohort study of infants born with a functional VAS inserted for severe cLUTO evaluated the perinatal and biochemical risk factors associated with advanced CKD stage 4–5, or eGFR <30 ml/min/1.73 m^2^, at 12 months of age.

In order to assess the potential benefit of any intervention, it is key that said intervention is operational. Since the inception of vesicoamniotic shunting more than four decades ago (22), multiple shunts have been used, including: double pig-tail (23), wire mesh (24) and double pig-tail plus double disk device (16). Dislocation and malfunction rates are dependent on the type of shunt, ranging from 20% to >80% for the simple pig-tail and wire mesh types (23, 24). These complication rates have marred the ability to understand the impact of in-utero shunting on infant kidney function. This study's use of the combined pig-tail + double disk device with a 100% functional rate in live-born infants offers the novel opportunity to investigate nephropathy in boys with severe cLUTO (16). Furthermore, the secure placement of this shunt type provided reliable urinary drainage after birth as well, allowing the stabilization and growth of these small and vulnerable neonates (25).

In addition to VAS functionality, the estimation of kidney function has not been standardized across publications, which can be misleading towards understanding outcomes. Some studies declare “normal renal function” without providing any definition (24, 26). Some rely only on SCr (27, 28), and others have omitted to show the data despite using a specific definition (29). In neonates with congenital anomalies of the kidney and urinary tract (CAKUT), including cLUTO, peak SCr is associated with discharge SCr (30). In our study, a neonatal peak SCr ≥2.0 mg/dl predicted CKD stage 4–5 at 1 year of age with a sensitivity of 100% and specificity of 83%. Subsequently, a neonatal discharge SCr ≥1.0 mg/dl was similarly predictive with the same sensitivity and specificity. It should be noted that the neonatal discharge SCr is a surrogate for kidney function when the infant is deemed clinically stable for parental care. Even if the neonatal length of stay or age varies between patients, this value should be less confounded by acute injury changes during intensive care. An additional finding in our study was the time to peak SCr, in which a longer rise in SCr for more than 6 days after birth was predictive of CKD stage 4–5 at 1 year of age. Clarifying the natural history of biomarker trends in this interventional population provides support to clinicians at the intensive care bedside in counseling parents. Ideally, neonatal SCr should decrease from the maternally-impacted value at birth. The trends identified in this study population show that most infants will have an increase in SCr, particularly those with poorer outcomes. Therefore, even if the initial SCr value is affected by maternal SCr, the time course and direction of change may now add further predictive value. Following hospital discharge, the nadir SCr in the first year has been recognized as predictive of more long-term kidney function at 1 and 2 years of age, in both retrospective and machine learning models (13, 31). In a mixed cohort of males and females with severe CAKUT, with and without fetal intervention, our group previously identified a nadir SCr > 0.6 mg/dl as predictive of CKD stage 3–5 over a range of follow-up from 7 months to 11.8 years (32). In a population of males with PUV, a nadir SCr ≥1 mg/dl was associated with CKD stage 3 or more, while a nadir SCr >0.85 mg/dl was predictive of CKD stage 2 or more at 1 year (9, 13). In our current cohort of males with severe cLUTO and VAS, a nadir SCr ≥0.5 mg/dl occurring in less than 160 days was associated with CKD stage 4–5 at 1 year with 100% sensitivity and 80% specificity. Infants with less advanced CKD at 12 months of age continued to experience a reduction in SCr beyond 5 months of age. The threshold of approximately 5 months of age is similar to that identified by Coleman and colleagues in a non-shunted group of boys with PUV only (13). Of note, in a different study of shunted patients with severe cLUTO, the authors defined normal kidney function as a SCr <0.5 mg/dl at 6 months of age (17). This definition, if adopted in our study, would have included infants who would be estimated to have CKD 2–3 at 1 year. This highlights the silence of nuance across scientific studies and the value of an understanding of natural history in clinical care.

As a biomarker independent of muscle mass and that does not cross the placenta, CysC is poised to be a more accurate measure of neonatal kidney function than SCr (19). In one study across a broad spectrum of congenital urologic obstructive pathologies, SCr was less reliable than CysC in estimating GFR throughout the first year, specifically in the cLUTO subgroup, due to poor infant weight gain and growth impacting the interpretation of SCr (33). In the previously referred to, mixed-sex CAKUT cohort, our group identified a neonatal CysC ≥3.0 mg/l associated with CKD stage 3–5 over long-term follow-up. Currently, in this all-male severe cLUTO group with successful VAS, a lower threshold neonatal CysC ≥2.5 mg/l was predictive of CKD stage 4–5 at 1 year of age. Though a single, early value of neonatal CysC was available for this study, future prospective analysis of serial CysC throughout the neonatal period as is more typical of SCr, may allow for the potential distinction of CysC as a stronger biomarker.

Natural trends in biomarkers are important for KRT planning, particularly in patient groups with high rates of end-stage kidney disease. A conservative approach with our cohort resulted in the neonatal discharge of the 14 survivors without a dialysis catheter, nor a dependence on KRT. In a retrospective analysis of PUV patients in the Pediatric Health Information System (PHIS) database, 56% of patients who had received a dialysis catheter did not go on to require kidney transplantation. Nearly three quarters of those had the catheter placed as a neonate (34), further supporting a thoughtful approach.

Only with a more detailed understanding of patient subpopulations and the impact of intervention can we better-define who may benefit from intervention and in what ways. Of the multiple published staging algorithms and scoring systems, fetal bladder volume is characterized as high-risk across a range from 5.4 to 35 to 40 cm^3^ (ml) (4, 17, 20). While our study included patients with a median volume of 39 cm^3^ at the highest end of that spectrum, the IQR reflects the inconsistency in these thresholds. Even more provocative has been the finding that the subgroup of patients with VAS showed a reduced sensitivity of the fetal intervention candidacy scoring system (17), which may suggest that the intervention changes the outcome or at least the character of the biomarker. Also variably understood, oligohydramnios is used to distinguish intervention candidates, while normal amniotic fluid volume in the second-trimester is nonetheless associated with ESKD by 2 years of age in up to one-third of cLUTO cases (35). This conflict stems from the oft-repeated conclusion that VAS improves survival rates by reducing pulmonary hypoplasia, without a protective effect on nephropathy (36). When improved selection criteria are cited as necessary for mitigating future kidney morbidity (22), perhaps the answers rest first in the fundamental functionality of the intervention.

Limitations of this study include its retrospective design and small sample size. Regarding the fetal urine analytes, analysis for this study was limited to measurement from a single, initial vesicocentesis based on the center protocol of the time, rather than from sequential sampling. While limited data suggests improved predictive capacity from serial measurements of fetal urine (37), the proteomic approach has been introduced on either single initial vesicocentesis (38) or even on amniotic fluid (39). Nonetheless, our sample size is comparable to that of other contemporary investigations of this rare disease and provides nuanced insight into the natural history of this growing population of children who have severe features of cLUTO and have undergone placement of VAS.

Future investigations should aim to expand the sample size by both number of subjects and centers. Given the vulnerability of cLUTO patients, a more specific understanding of non-invasive biomarkers would also improve care. The prospective trending of the urine protein profile and/or the peptidomic-metabolomic signature can be done with little to no discomfort to the infant. Ultrasonographic parameters, including renal parenchymal area previously studied by our group in this specific population (40), are also intriguing for expanded patient-friendly study across multiple centers. As the medical community better defines patient populations through staging algorithms, and recognizing the protracted chronicity of obstructive nephropathy, it has become inadequately vague to counsel a family with the generalization that one third of cLUTO survivors will need KRT (41, 42). In the spirit of precision medicine, we should aim to clarify the early characteristics of patients with cLUTO in whom KRT may be indicated.

## Data Availability

The raw data supporting the conclusions of this article will be made available by the authors, without undue reservation.
